# AICAR and nicotinamide treatment synergistically augment the proliferation and attenuate senescence-associated changes in mesenchymal stromal cells

**DOI:** 10.1186/s13287-020-1565-6

**Published:** 2020-02-03

**Authors:** Mohammadhossein Khorraminejad-Shirazi, Mahsa Sani, Tahereh Talaei-Khozani, Mohammadreza Dorvash, Malihe Mirzaei, Mohammad Ali Faghihi, Ahmad Monabati, Armin Attar

**Affiliations:** 1 Student Research Committee, Shiraz University of Medical Sciences, Shiraz, Iran; 2 Cell and Molecular Medicine Student Research Group, School of Medicine, Shiraz University of Medical Sciences, Shiraz, Iran; 3 Tissue Engineering Department, School of Advanced Medical Science and Technology, Shiraz University of Medical Science, Shiraz, Iran; 4 Tissue Engineering Lab, Department of Anatomical Sciences, School of Medicine, Shiraz University of Medical Sciences, Shiraz, Iran; 5 Pharmaceutical Sciences Research Center, Shiraz University of Medical Sciences, Shiraz, Iran; 6 Persian BayanGene Research and Training Center, Shiraz University of Medical Sciences, Shiraz, Iran; 7 Center for Therapeutic Innovation, Department of Psychiatry and Behavioral Sciences, University of Miami Miller School of Medicine, Miami, FL USA; 8 Department of Pathology, School of Medicine, Shiraz University of Medical Sciences, Shiraz, Iran; 9 Hematology Research Center, Shiraz University of Medical Sciences, Shiraz, Iran; 10 Department of Cardiovascular Medicine, Shiraz University of Medical Sciences, PO Box 71344-1864, Shiraz, Iran

**Keywords:** mTOR, AMPK, SIRT1, Autophagy, Senescence, AICAR, Nicotinamide, Mesenchymal stromal cell

## Abstract

**Background:**

Mesenchymal stromal cell (MSC) stemness capacity diminishes over prolonged in vitro culture, which negatively affects their application in regenerative medicine. To slow down the senescence of MSCs, here, we have evaluated the in vitro effects of 5-aminoimidazole-4-carboxamide ribonucleotide (AICAR), an AMPK activator, and nicotinamide (NAM), an activator of sirtuin1 (SIRT1).

**Methods:**

Human adipose-derived MSCs were cultured to passage (P) 5. Subsequently, the cells were grown in either normal medium alone (control group), the medium supplemented with AICAR (1 mM) and NAM (5 mM), or in the presence of both for 5 weeks to P10. Cell proliferation, differentiation capacity, level of apoptosis and autophagy, morphological changes, total cellular reactive oxygen species (ROS), and activity of mTORC1 and AMPK were compared among different treatment groups.

**Results:**

MSCs treated with AICAR, NAM, or both displayed an increase in proliferation and osteogenic differentiation, which was augmented in the group receiving both. Treatment with AICAR or NAM led to decreased expression of β-galactosidase, reduced accumulation of dysfunctional lysosomes, and characteristic morphologic features of young MSCs. Furthermore, while NAM administration could significantly reduce the total cellular ROS in aged MSCs, AICAR treatment did not. Moreover, AICAR-treated cells possess a high proliferation capacity; however, they also show the highest level of cellular apoptosis. The observed effects of AICAR and NAM were in light of the attenuated mTORC1 activity and increased AMPK activity and autophagy.

**Conclusions:**

Selective inhibition of mTORC1 by AICAR and NAM boosts autophagy, retains MSCs’ self-renewal and multi-lineage differentiation capacity, and postpones senescence-associated changes after prolonged in vitro culture. Additionally, co-administration of AICAR and NAM shows an additive or probably a synergistic effect on cellular senescence.

## Background

After a prolonged in vitro culture of mesenchymal stromal cells (MSC), aging, which is a functional decline of the cells’ proliferation rate and skewed multi-lineage differentiation capacity, happens [1, 2]. Continuous in vitro culture of MCSs ultimately causes undesirable outcomes and reduced efficacy of MSC-based therapies [3–5]. Furthermore, aged MSCs lose their capacity to differentiate toward osteogenic lineage [6, 7].

The decline in the stem cell function and properties during prolonged culture might be due to the deregulation of the mechanistic target of rapamycin complex 1 (mTORC1) and 5′-adenosine monophosphate-activated protein kinase (AMPK) signaling pathways. Hyperactivation of mTOR signaling has a pivotal role in cellular senescence [8–10], derivation of differentiation [11], and depletion of stem cell pool [12]. Rapamycin, an inhibitor of mTORC1, has been used in several studies to maintain function and growth, and retard cellular senescence of various types of stem cells [11–14].

Another underlying factor in the pathogenesis of aging is the production of reactive oxygen species (ROS) [11, 15]. Hyperactivation of mTOR can also lead to increased intracellular ROS generation and cellular damage [16]. Additionally, high levels of mTORC1 activity can negatively regulate autophagy in the stem cells, leading to the accumulation of dysfunctional organelles [17]. Failure of mitophagy, the autophagy of mitochondria, causes a surge in ROS generation and ROS-mediated injuries [18, 19]. Activation of the sirtuins family of NAD^+^-dependent deacetylases can boost mitophagy and antioxidant mechanisms in cells [20, 21]. Increased intracellular NAD^+^ to NADH ratio activates the sirtuin1 (SIRT1) and, in turn, raises the AMPK activity. AMPK then phosphorylates the regulatory-associated protein of TOR (Raptor) on Ser722/Ser792 to attenuate the mTORC1 activity [22, 23]. Nicotinamide (NAM), which is converted to NAD^+^ in cells, can enhance the in vitro replication and multi-lineage differentiation capacity of human bone marrow stem cells through activation of SIRT1 [24]. Also, NAM can prevent the senescence of human primary keratinocytes and increase their proliferative potential [25].

In this study, to attenuate the aging of the MSCs, 5-aminoimidazole-4-carboxamide ribonucleotide (AICAR), an AMPK activator [26], and NAM were employed. We studied the effects of AICAR and NAM on the replicative capacity, senescence-associated changes, and osteogenic differentiation potential of MSCs after prolonged in vitro culture. Furthermore, we evaluated the effects of AICAR and NAM on AMPK and mTORC1 activity and autophagy. We also investigated whether the simultaneous administration of AICAR and NAM would result in an additive or synergistic effect on cellular senescence compared with single compound treatment (either AICAR or NAM).

## Materials and methods

### Isolation and culture of human adipose-derived MSCs

Human MSCs were isolated from the adipose tissue of three different healthy individuals, and informed consent was obtained according to the Shiraz University of Medical Sciences ethics committee guidelines [27, 28]. Initially, fragments of human adipose tissue were washed with phosphate-buffered saline (PBS) to remove the contaminating hematopoietic cells; then, they were minced and digested with 0.2% collagenase type I (Gibco, Paisley, UK) at 37 °C. Then, the cell suspension was centrifuged, and the pellets comprising the adherent stromal cells were cultured in medium containing Dulbecco’s modified Eagle’s medium (DMEM) (Gibco), penicillin (100 U/ml), streptomycin (100 mg/ml) (Sigma-Aldrich, St. Louis, MO, USA), GlutaMAX (2 mM) (Invitrogen, Paisley, UK), and 10% fetal bovine serum (FBS) (Gibco) and maintained in 5% CO_2_ at 37 °C for 5 weeks to passage (P) 5. During this stage, the cell medium was changed after 48 h, the floating cells were removed, and the cells were sub-cultured in MSC growth medium.

Subsequently, MSCs were seeded at a density of 500,000 in T-75 flasks and grown in normal medium alone (control group), medium supplemented with AICAR at 1 mM (Sigma-Aldrich) or NAM at 5 mM (Sigma-Aldrich), or in the presence of both AICAR and NAM (1 mM and 5 mM, respectively). Cell cultures were maintained in the continuous culture for about 5 weeks for a further five passages (P10).

### Immunophenotyping of adipose-derived MSCs

To characterize the adipose-derived MSCs, we harvested the cells at P3 by trypsin/EDTA. Adipose MSCs were identified for the surface markers CD45, CD34, CD144, CD44, and CD90 by flow cytometry using Flow Cytometer laser 488 nm (Becton Dickinson, NJ, USA) and analyzed with FlowJo™ Software.

### Cell viability assay and doubling time

The 3-(4,5 dimethyl-2-thiazolyl)-2,5-diphenyl tetrazolium bromide (MTT) assay (Cat# M5655; Sigma-Aldrich) was performed on days 3 and 7 after cell seeding to evaluate the proliferation of MSCs at P10. Briefly, 500 μl of 0.5 mg/ml 3-(4,5 dimethyl-2-thiazolyl)-2,5-diphenyl tetrazolium bromide was added to each study group, and the cells were incubated for 4 h at 37 °C. Then, the MTT solution was removed, and 200 μl of DMSO (Merck) was added to each well. The optical densities (ODs) of the stained solutions were measured with POLARstar Omega Plate Reader Spectrophotometer at 570 nm wavelength [29].

Doubling time was calculated at passages 5, 6, 8, and 10 by following the formula:

$$ \mathrm{Doubling}\ \mathrm{time}=\mathrm{duration}\times \log\ (2)/\log\ \left(\mathrm{final}\ \mathrm{concentration}\right)-\log\ \left(\mathrm{initial}\ \mathrm{concentration}\right) $$

### Morphological assessment of senescent cells

Phase-contrast light microscopic images were taken from each study group. To evaluate the morphology of the MSCs on the tissue culture dish at passage 10, we used ImageJ software (NIH, MD, USA). Briefly, a line was drawn over the scale bar, using the “Straight free hand line,” and under the “Analyze” tab, “Set Scale” was selected. In the “Set Scale” window, the length of the known distance of the scale bar and units of measurement (500 μm in our study) was typed in the appropriate boxes. Using the “Freehand selection” tool, we drew a line on the outline of each cell. Then, under the “Analyze” tab, “Measure” was selected to calculate the cross-sectional surface area of the selected cell. This procedure was performed for at least 100 cells per study group (AICAR, NAM, AICAR+NAM, and control).

### β-Galactosidase activity assessment

Cellular senescence assay was performed using the senescence cells histochemical staining kit (Cat# CS0030, Sigma-Aldrich) according to the manufacturer’s instructions. In short, the cells were incubated with 1× fixation buffer for 7 min at room temperature. Next, the cells were washed in PBS and stained with a staining mixture (containing X-gal) at 37 °C overnight. The cells were observed under a bright-field microscope (× 200 magnification), and the percentage of β-galactosidase-positive (SA-β-gal) cells was measured by counting the blue-stained cells and the total number of cells (the total number of 100 cells was counted for each treatment group).

### Acridine orange staining

To assess the lysosomal membrane integrity and distribution of nucleic acids within the intracellular compartments, we stained the cells with Acridine Orange. MSCs were stained with 10 μg/ml of Acridine Orange (BDH stains, UK) for 30 min at 37 °C, washed with media, and assessed using an Olympus BX53 fluorescent microscope. When highly concentrated in acidic lysosomes with an intact membrane, Acridine Orange emits red fluorescence. At lower concentrations, i.e., when bound to double-stranded DNA in the cytoplasm and nucleus, Acridine Orange emits green fluorescence [11].

### Flow cytometric analysis of cellular ROS

Accumulation of cellular ROS was measured at P5 and the end of P10, after incubation with AICAR, NAM, and both AICAR and NAM. The level of cytoplasmic ROS was evaluated with reactive oxygen species detection assay kit (Abcam, Cambridge, MA, USA). Following the manufacturer’s instruction, the cells were washed with PBS, suspended, and stained in a conical test tube with 20 μM 2′,7′-dichlorofluorescin diacetate (DCFDA) in the supplemented buffer (10% FBS in the buffer 1X) and incubated for 30 min at 37 °C in the dark. DCFDA is changed by intracellular ROS into 2′, 7′-dichlorofluorescein (DCF), a highly fluorescent compound. DCF is excited by the 488-nm laser and detected at 535 nm. The measurement of ROS production was monitored immediately by Flow Cytometer laser 488 nm (Becton Dickinson, NJ, USA). A total of 10,000 cells were analyzed for each sample, and data analysis was performed using FlowJo™ Software.

### Differentiation potential

#### Osteogenic differentiation

Adipose-derived MSCs at P8 were seeded at a density of 30,000 in 24-well plates. After overnight incubation in MSC growth media, the culture media were replaced with osteogenic induction medium composed of 0.1 mM dexamethasone, 50 μM ascorbic acid, 10 mM glycerol phosphate (Sigma-Aldrich), and 10% FBS (Gibco) in DMEM. In this stage, the cells were grown in osteogenic induction medium alone (control group) or osteogenic induction medium supplemented with AICAR at 1 mM or NAM at 5 mM or in the presence of simultaneous AICAR and NAM (1 mM and 5 mM, respectively).

To further clarify our method, initially, we used P10 MSCs for osteogenic differentiation. However, the MSCs of the control group failed to differentiate. In our second effort with the cells of the control group, we found most of them detached from the culture plate. None of our efforts for the differentiation of P9 MSCs was successful. Following that, and for the sake of reasonable comparison between the treatment groups and the control group, we decided to use P8 MSCs.

After incubation for 28 days, to assess osteogenic differentiation, RNA was isolated and mRNA expression of the markers of osteogenic differentiation—osteopontin, Runt-related transcription factor 2 (Runx-2), and alkaline phosphatase (ALP)—was determined by quantitative reverse transcription polymerase chain reaction (qRT-PCR). Also, to detect mineralized matrix, we washed the cells with PBS, fixed them with 4% paraformaldehyde for 15 min, and then washed them with distilled water. Following that, to detect the extracellular calcium deposits, we stained the cells with Alizarin Red S (Sigma-Aldrich) for 20 min. For quantification, the Alizarin Red S was eluted by 5% sodium dodecyl sulfate in 0.5 N HCL for 15 min, and the optical density was evaluated at 405 nm, using NanoDrop 2000c Spectrophotometer (Thermo Fisher Scientific) [11].

#### Adipogenic differentiation

Adipose-derived MSCs at P8 were seeded at a density of 30,000 in 24-well plates. After overnight incubation in MSC growth media, the culture media were replaced with AdipoMAX™ Differentiation Media (Sigma-Aldrich) to induce adipogenic differentiation. At this point, MSCs were grown in AdipoMAX™ Differentiation Media alone (control group) or AdipoMAX™ differentiation Media supplemented with AICAR at 1 mM or NAM at 5 mM or in the presence of simultaneous AICAR and NAM (1 mM and 5 mM, respectively).

To clarify, P10 and P9 MSCs failed to successful adipogenic differentiation. Thus, for a reasonable comparison between the treatment groups and the control group, we decided to use P8 MSCs for adipogenic differentiation.

After incubation for 21 days, to evaluate adipogenesis, RNA was isolated and mRNA expression of markers of adipogenic differentiation—lipoprotein lipase (LPL) and peroxisome proliferator-activated receptor γ (PPAR-γ)—was determined by qRT-PCR. Moreover, the accumulation of the intracellular lipid was quantified following staining with Oil Red O (Sigma-Aldrich). Briefly, the cells were fixed with 4% paraformaldehyde in PBS for 15 min and stained with 0.5% Oil Red O in isopropanol for 15 min. Then, the cells were washed with PBS and observed under the microscope for visualization. Stained oil droplets were dissolved in isopropanol for 15 min, and absorbance at 518 nm was evaluated, using NanoDrop 2000c Spectrophotometer (Thermo Fisher Scientific) [24].

### qRT-PCR analysis

The total RNA was extracted from the cultured cells, using the GeneJET RNA Purification Kit (Thermo Fisher Scientific) according to the company’s instructions. The quantity and purity of RNA were evaluated with an Epoch microplate spectrophotometer, and cDNA was synthesized using a RevertAid First Strand cDNA Synthesis Kit (Thermo Fisher Scientific) according to the manufacturer’s protocol. qRT-PCR was performed, using the prepared cDNA and the corresponding primers for Osteopontin, Runx-2, ALP, LPL, and PPAR-**γ**, P16, and P21. qRT-PCR was performed on a QIAGEN Rotor-Gene Q system using RealQ Plus Master Mix Green (Ampliqon). PCR conditions were as follows: 5 min of initial denaturation at 95 °C, followed by 35 cycles of denaturation at 94 °C for 30 s, annealing was conducted at 60 °C for 30 s, followed by 30 s extension at 72 °C, and 5 min final extension at 72 °C. PCR results were quantitatively analyzed by Rotor-Gene Q Series Software. The relative quantification of our desired mRNAs was determined, using the 2^−ΔΔCT^ method, and GAPDH was used as a housekeeping gene. Primer sequences will be provided upon request.

### Immunofluorescence staining

MSCs were cultured overnight on glass slides and fixed at room temperature with 4% paraformaldehyde for 20 min. Fixed MSCs were washed three times with PBS and permeated and blocked with PBS containing 10% goat serum, 1% BSA, and 1% Triton X100 for 45 min at room temperature. After washing the cells with PBS, MSCs were incubated at room temperature for 60 min with primary antibody against anti-AMPK alpha 1 (phospho T183) + AMPK alpha 2 (phospho T172) (Cat# ab133448, Abcam, 1:100), phospho-p70 S6 Kinase (Thr389) (Cat# 9205, Cell Signaling Technology, 1;100), anti-LC3B(Cat# ab192890, Abcam, 1 μg/ml), anti-Bcl-2 (Cat# ab7973, Abcam, 1:200), anti-Bax (Cat# ab5714, Abcam, 1:200), anti-caspase-3 (Cat# MAB10753; Sigma Aldrich, 1:100) diluted in PBS containing 1% goat serum, 0.1% BSA, and 0.05% Tween 20. Subsequently, the cells were washed with PBS and incubated at room temperature with respective fluorescent-conjugated secondary antibodies (Cat# F2772*,* Sigma-Aldrich 1:160, and Cat# SC-2090, Santa Cruz Biotechnology, 1:100) for 45 min in the dark. The nuclei were counterstained with 4,6-diamino-2-phenylindole dihydrochloride (DAPI; Millipore Cat #S7113, 1:1000). The specimens were visualized under an Olympus BX53 fluorescent microscope [30].

### Annexin V staining

To assess the cellular apoptosis, we used Annexin V-FITC Apoptosis Detection Kit (Abcam) according to the manufacturer’s protocol. Briefly, the cells were washed with media and harvested using trypsin-EDTA (Gibco). Then, 5 × 10^5^ cells were collected and incubated with Annexin V-FITC for 5 min at room temperature in the dark. Afterward, the cells were detected by flow cytometry using Flow Cytometer laser 488 nm (Becton Dickinson, NJ) and analyzed with FlowJo™ Software [31].

### Data analysis

Data are presented as mean ± standard deviation (SD). For quantitative analysis of the differences among the mean values between the groups, data were analyzed using one-way analysis of variance (ANOVA) with Turkey’s post hoc multiple comparison test through GraphPad Prism software (version 8.0.1; CA, USA). All experiments were performed at least in triplicate. A value of *p* < 0.05 was considered statistically significant.

## Results

### MSC culture

Two days after the initial seeding, the isolated adipose-derived MSCs were attached to the plates demonstrating fusiform-like appearance and became confluent after 12–18 days. The cells were maintained in 5% CO_2_ at 37 °C for 5 weeks to P5. Following that, MSCs at P5 were divided into four groups of AICAR, NAM, AICAR+NAM, and control groups and were treated with AICAR (1 mM), NAM (5 mM), and AICAR+NAM (1 mM and 5 mM, correspondingly) and in the absence of any of the mentioned compounds for further five passages.

### MSC characteristics

The isolated adipose-derived MSCs at P3 were harvested by trypsin/EDTA and were negative for the surface markers CD45, CD34, and CD144 and positive for the markers CD44 and CD90 by flow cytometry (Table 1).

**Table 1:** Adipose tissue-derived mesenchymal stromal cells markers expression

CD 44	CD 90	CD 34	CD 144	CD 45
92.31% ± 1.05	94.74% ± 0.93	1.69% ± 0.87	1.185% ± 0.44	2.22% ± 0.15

### Population doubling time assay

**Fig. 1:**
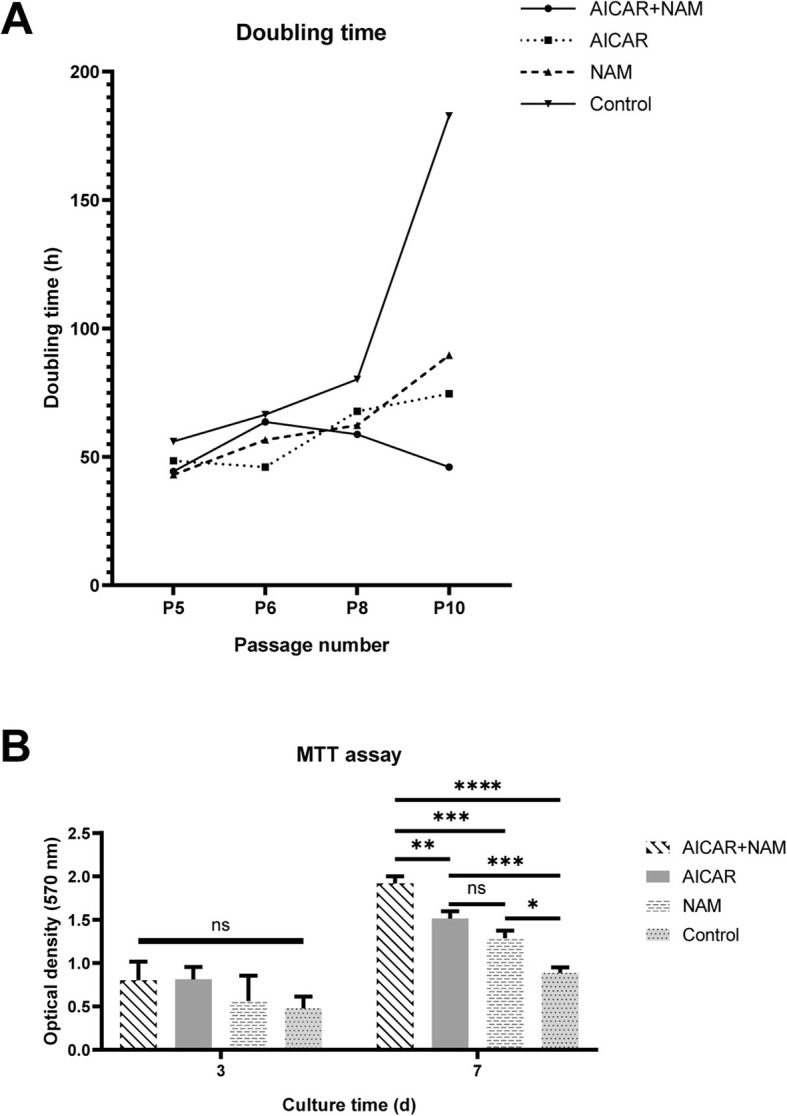
Effect of AICAR and NAM on doubling time and MTT assay of the aging MSCs. **a** AICAR, NAM, and combination of AICAR+NAM reduce the doubling time of MSCs compared to the control group, from P5 to P10. **b** Proliferative capacity of MSCs at P10 was determined by MTT assay at the third and seventh day of culture (*n =* 3 independent experiments). Each bar indicates mean ± SD (*p < .05; **p < .01; ***p < .001; ****p < .0001). Abbreviations: AICAR, 5-aminoimidazole-4-carboxamide ribonucleotide; NAM, nicotinamide; MTT, 3-[4,5-dimethylthiazol-2-yl]2,5-diphenyl tetrazolium bromide

As demonstrated in Fig. 1a, MSCs in the untreated control group had a steeper increase in doubling time, whereas cells cultured in the presence of either NAM or AICAR had a lower doubling time after long-term in vitro expansion. Of note, MSCs cultured in the presence of both NAM and AICAR had the lowest doubling time at P10 compared to that of the other groups.

### MTT assay

To examine the effects of AICAR and NAM on the proliferation of MSCs, we estimated the cell numbers in each treatment group by MTT assay (Fig. 1b). MSCs of different study groups at P10 were harvested, and MTT assay was done on the third and seventh days after seeding. Although compared to the control group, there was no significant increase in the estimated number of cells after 3 days, the cell proliferation after 7 days was dramatically increased with concomitant use of AICAR and NAM or each compound alone (AICAR or NAM) compared to the control group.

### Expression of cyclin-dependent kinase inhibitors

We also observed a decline in the expression of genes associated with cell cycle progression and cellular senescence [32]. Notably, cells treated with AICAR+NAM showed a reduction in the expression of *P16* and *P21* genes compared to the control group (Additional file 1).

### Cell morphology

**Fig. 2:**
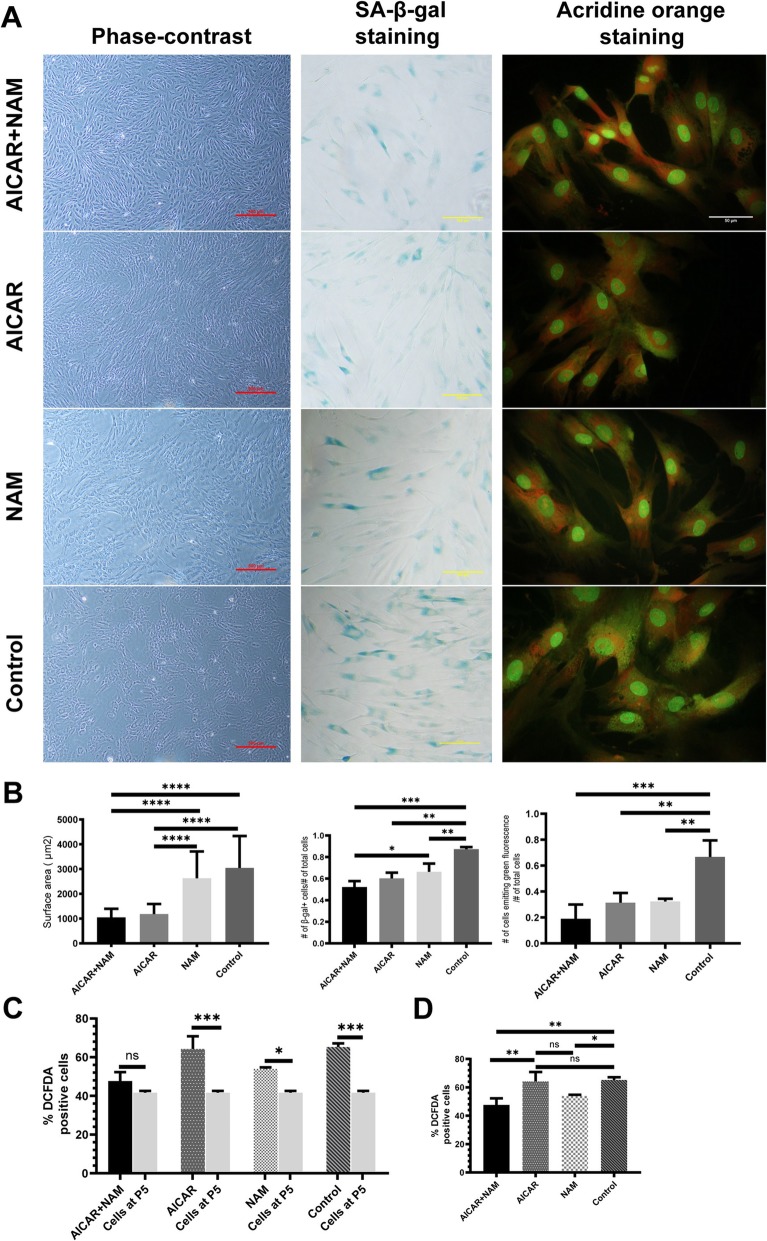
Distinct effects of AICAR, NAM, and concomitant AICAR+NAM treatment on senescence-associated changes of MSCs and total cellular reactive oxygen species (ROS). MSCs at passage 5 were treated with AICAR, NAM, and AICAR+NAM for further five passages. **a** Phase-contrast images of MSCs (P10) (scale bar = 500 μm), SA-β-gal expression, visualized using light microscopy (scale bar = 100 μm), and fluorescent micrograph (scale bar = 50 μm) of the Acridine Orange stained MSCs at P10 of the four groups. **b** Left panel: the surface area of the MSCs (P10), calculated using ImageJ software, indicates that cells treated with AICAR alone or AICAR+NAM displayed a significantly lower cross-sectional surface area compared to the NAM-treated cells and the untreated group. Middle panel: prevalence of the SA-β-gal-positive cells, calculated as the number of blue cells per the total number of cells counted. Our data show that treatment with AICAR and NAM reduces the expression of SA-β-gal. Right panel: prevalence of senescent cells determined by the number of green fluorescence-emitting cells per the total number of cells counted. Untreated cells displayed the highest frequency of cells emitting green fluorescence and the least frequency of red fluorescence-emitting cells, compared to the treatment groups. Each bar indicates mean ± SD. **c** Total cellular ROS was measured at P5 and P10 by staining with DCFDA, followed by flow cytometry analysis (*n =* 3 independent experiments). **d** Comparison between total cellular ROS of the study groups at P10 (*n =* 3 independent experiments). Each bar indicates mean ± SD (*p < .05; **p < .01; ***p < .001; ****p < .0001). Abbreviations: SA-β-gal, senescence-associated β-galactosidase; ROS, reactive oxygen species; DCFDA, 2′,7′-dichlorofluorescin diacetate; AICAR, 5-aminoimidazole-4-carboxamide ribonucleotide; NAM, nicotinamide

To evaluate the morphology of MSCs, the surface area of the cells was determined, using the technique described in the “Materials and methods” section. MSCs cultured with concomitant use of AICAR and NAM showed the morphology of MSCs in their youth with small, spindle-like shape and low cytoplasmic granularity, whereas MSCs in the control group displayed characteristic features of senescent MSCs [32] with their flattened and enlarged morphology and granular cytoplasm. Of note, MSCs treated with AICAR alone showed the characteristic morphology of young MSCs, like the AICAR+NAM group, while NAM-treated cells exhibited morphological features of senescent MSCs (Fig. 2a, b).

### SA-β-gal expression

Cells in the control group have an increased percentage of SA-β-gal-positive cells after extensive culture, as it is expected from the senescent cells [32], compared to MSCs treated with AICAR, NAM, and combined AICAR+NAM. The cells treated with NAM alone expressed SA-β-gal more than the AICAR+NAM group (Fig. 2a, b).

### Acridine Orange staining

Untreated MSCs in the control group showed an increase in green fluorescence and a decrease in red fluorescence when stained with Acridine Orange, suggesting altered lysosome function and senescence [33], while treatment with AICAR, NAM, and AICAR+NAM protected the cells against such degenerative alternations (Fig. 2a, b).

### Total cellular ROS generation

Levels of cytoplasmic ROS were evaluated by DCFDA at P5 (before incubation with any of the compounds) and P10 (after continuous in vitro culture in the presence of AICAR, NAM, and concomitant AICAR+NAM). Simultaneous use of AICAR and NAM reduced cytoplasmic ROS as there was no significant change in the levels of ROS at P10 in comparison with P5. The untreated cells in the control group and AICAR-treated cells showed a dramatic rise in intracellular ROS. Additionally, NAM-treated MSCs displayed a rise in cytoplasmic ROS after prolonged culture at P10 compared to MSCs at P5 (Fig. 2c). To further elucidate the effect of administration of AICAR, NAM, and AICAR+NAM on ROS generation, we compared the cytoplasmic ROS of the different treatment groups to each other at P10 (Fig. 2d). Combined AICAR+NAM treatment displayed the lowest amounts of ROS, with no significant difference with the NAM group. It is of note that there was no substantial difference in the cytoplasmic ROS of the cells treated with AICAR alone and the untreated cells in the control group at P10. Altogether, our data showed that the use of NAM, either as AICAR+NAM or NAM alone, lowered the ROS generation within the cells, while treatment with AICAR alone did not considerably affect the cytoplasmic ROS after long term in vitro aging.

### Immunofluorescence staining of apoptotic markers

**Fig. 3:**
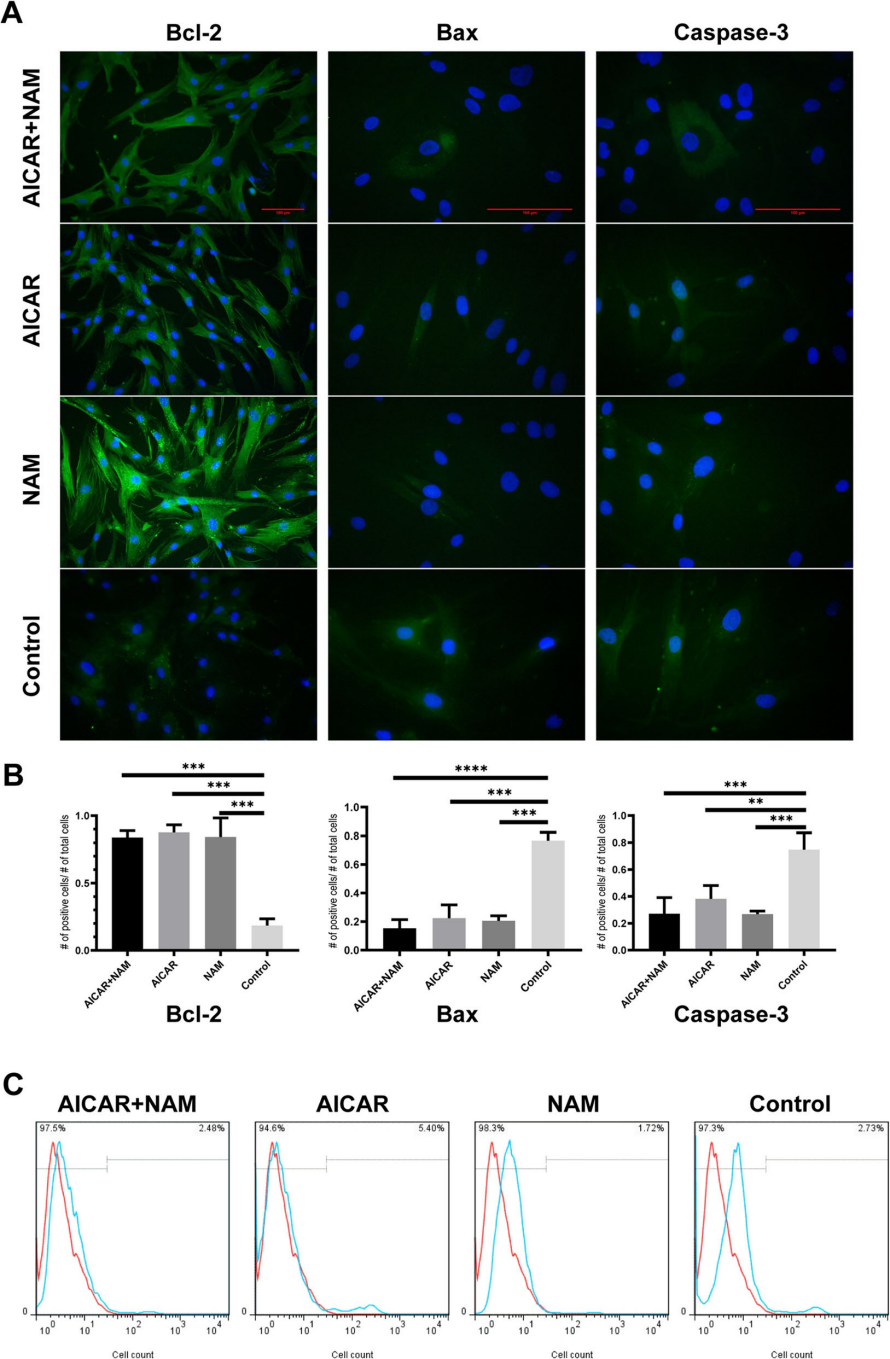
Effects of AICAR, NAM, and AICAR+NAM treatment on markers of apoptosis and cellular apoptosis. **a** Immunofluorescence staining of the study groups at P10, for Bcl-2, Bax, and Caspase-3. The nuclei were visualized using DAPI (blue fluorescence). AICAR and NAM treatments decrease pro-apoptotic proteins, Bax, and Caspase-3 and increase the anti-apoptotic protein, Bcl-2 (scale bar = 100 μm). **b** Representative data of Bcl-2, Bax, and Caspase-3 immunofluorescence, calculated as the number of positive cells per the total number of cells. Each bar indicates mean ± SD. **c** Flow cytometry analysis of Annexin V assay (*p < .05; **p < .01; ***p < .001; ****p < .0001). Abbreviations: AICAR, 5-aminoimidazole-4-carboxamide ribonucleotide; NAM, nicotinamide

MSCs that were concomitantly treated with AICAR+NAM or any of the compounds (AICAR or NAM) alone displayed an increase in anti-apoptotic protein Bcl-2 and a decrease in pro-apoptotic proteins, Bax and Caspase-3 (Fig. 3a, b), compared to untreated control cells. Notably, the cells treated with AICAR alone had the highest Caspase-3 immunofluorescence-positive cells among the treatment groups.

### Annexin V flow cytometry assay

Our data showed that cells treated with AICAR had the highest apoptosis rate, almost two times that of the untreated cells. In detail, the percentage of Annexin V-positive cells was 2.73% in the untreated cells compared to 5.4% in the AICAR-treated cells, 1.72% in the NAM-treated cells, and 2.48% in the AICAR+NAM group (Fig. 3c).

### Multi-lineage differentiation potential

**Fig. 4:**
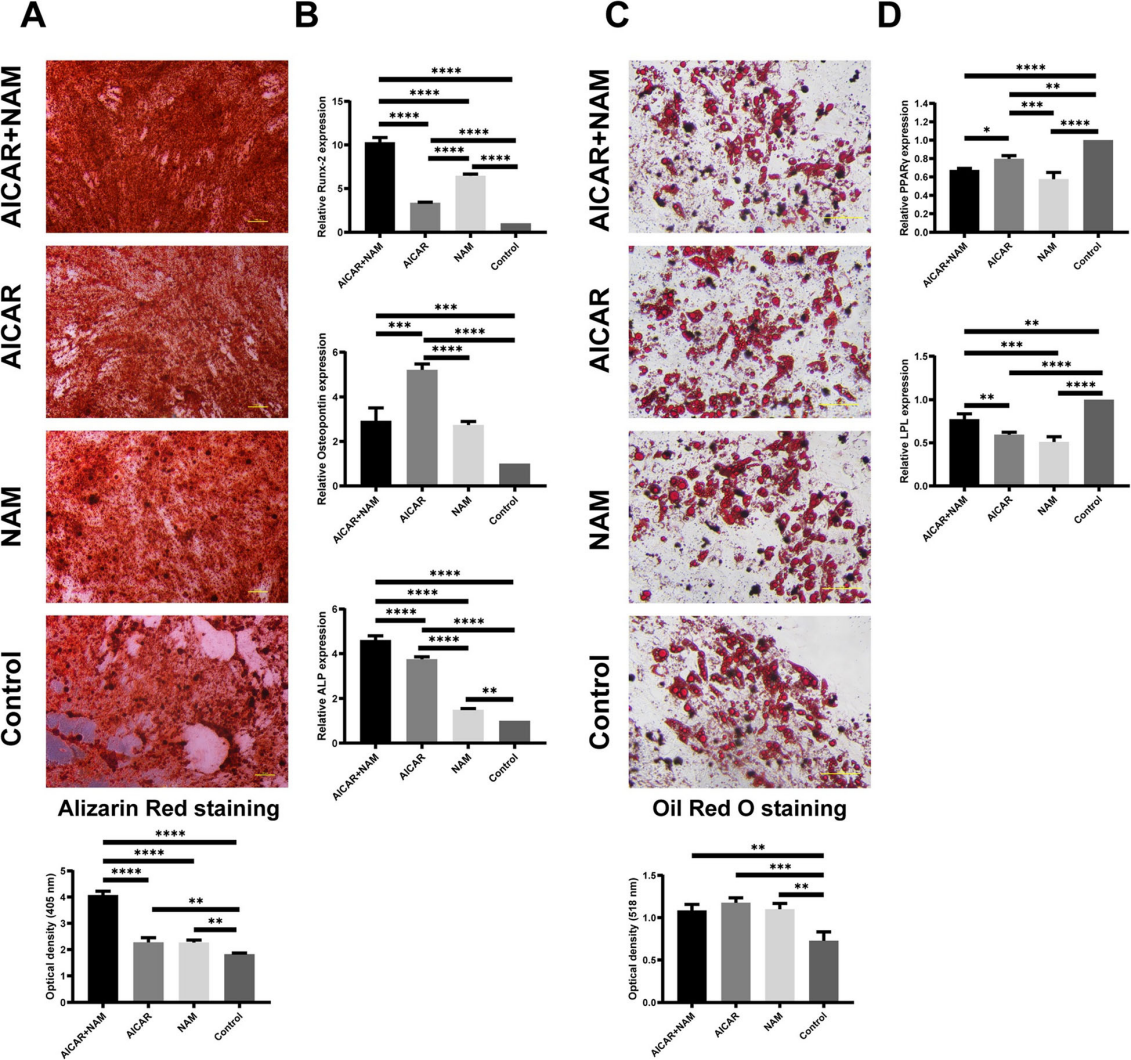
Effect of AICAR and NAM treatment on differentiation potential of MSCs after long-term in vitro culture. MSCs at P8 were cultured in osteogenic or adipogenic induction media in the presence of AICAR (1 mM), NAM (5 mM), or AICAR+NAM (1 mM and 5 mM, respectively) or in the absence of AICAR and NAM (control group). **a** Cells were stained with Alizarin Red S, and the accumulation of calcium deposits was visualized using light microscopy (scale bar = 100 μm) and quantified by spectrophotometry (*n =* 3 independent experiments). Each bar indicates mean ± SD. **b** Expression of markers of osteogenesis—Runx-2, osteopontin, and ALP—was determined by qRT-PCR analysis (*n =* 3 independent experiments). Each bar indicates mean ± SD. **c** For the evaluation of adipogenesis, cells were stained with Oil Red O, and lipid accumulation was visualized using light microscopy (scale bar = 100 μm) and quantified by spectrophotometry (*n =* 3 independent experiments). Each bar indicates mean ± SD. **d** mRNA expression of markers of adipogenesis—LPL and PPAR-γ—was determined by qRT-PCR analysis (*n =* 3 independent experiments). Each bar indicates mean ± SD (*p < .05; **p < .01; ***p < .001; ****p < .0001). Abbreviations: Runx-2, Runt-related transcription factor 2; ALP, alkaline phosphatase; LPL, lipoprotein lipase; PPAR-γ, peroxisome proliferator-activated receptor γ; AICAR, 5-aminoimidazole-4-carboxamide ribonucleotide; NAM, nicotinamide

We also studied the effect of AICAR and NAM treatment on the differentiation potential of MSCs after a long-term in vitro culture using adipogenic and osteogenic induction media. We observed that higher levels of matrix mineralization were achieved following treatment with AICAR and NAM. Noticeably, the treatment of cells with simultaneous AICAR+NAM significantly increased the calcium deposition (Fig. 4a). Moreover, the expression of the markers of osteogenesis—Runx-2, osteopontin, and ALP—was enhanced in AICAR+NAM-, AICAR only-, and NAM only-treated cells compared to the untreated cells (Fig. 4b). Further, we detected a greater level of lipid accumulation in our treatment groups compared to the control group (Fig. 4c). Also, the expression of LPL and PPAR-γ mRNAs was decreased after treatment with AICAR and NAM (Fig. 4d).

Overall, our data indicate that the treatment of MSCs with AICAR and NAM maintained and improved their multi-lineage differentiation capacity, which otherwise deteriorated after a long-term in vitro culture.

### Immunofluorescence staining of AMPK, p70S6K, and LC3B

**Fig. 5:**
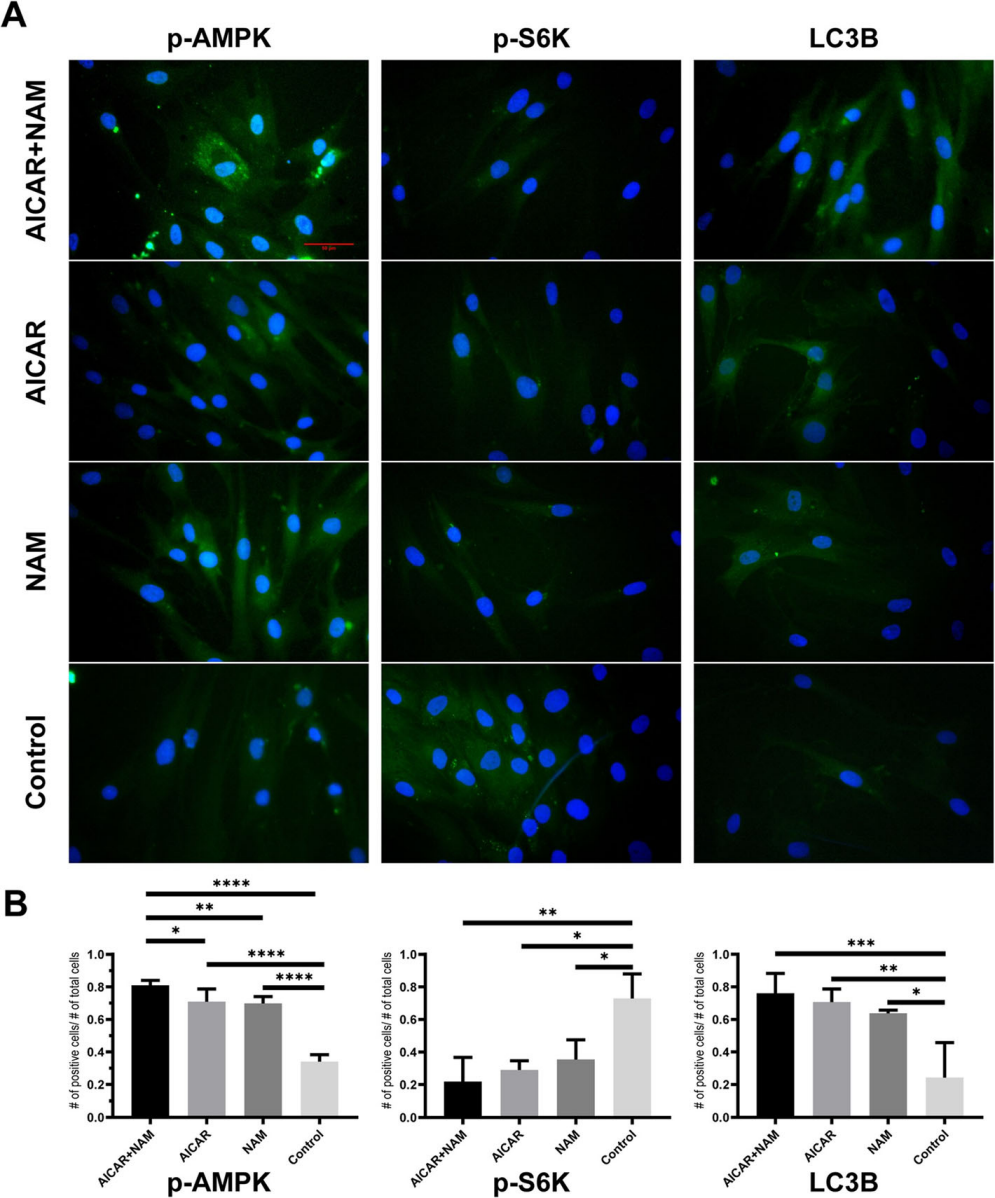
Effects of AICAR, NAM, and AICAR+NAM on the intracellular signaling pathways of AMPK, mTORC1, and autophagy. **a** MSCs at passage 5 were treated with AICAR, NAM, and AICAR+NAM for further five passages. Immunofluorescence staining of the study groups at P10 using anti-phospho-AMPK (AMPK-α1-pThr183 and AMPK-α2-pThr172) antibody, anti-phospho-p70 S6 Kinase (Thr389) antibody, and anti-LC3B antibody. The nuclei were visualized using DAPI (scale bar = 100 μm). **b** Representative data of phospho-AMPK, Phospho-p70 S6 kinase (Thr389), and LC3B immunofluorescence, calculated as the number of positive cells per total number of cells. Each bar indicates mean ± SD (*p < .05; **p < .01; ***p < .001; ****p < .0001). Abbreviations: AICAR, 5-aminoimidazole-4-carboxamide ribonucleotide; NAM, nicotinamide

To determine the intracellular mechanisms tangled in preserved proliferation and differentiation capacities of MSCs in different treatment groups, we studied the responses of intracellular signaling cascades to AICAR and NAM. The cells exposed to any of the compounds alone (AICAR or NAM) and combined NAM and AICAR showed higher levels of AMPK activity as determined by the use of anti-AMPK alpha 1 (phospho T183) + AMPK alpha 2 (phospho T172) antibody compared to the untreated control group (Fig. 5a, b). The activity of mTORC1 was calculated by measuring its phosphorylated downstream effector, S6 kinase 1 (S6K1). MSCs in all the treatment groups showed a reduction in p-S6K1 compared to the control group (Fig. 5a, b). Autophagosome formation and level of cellular autophagy were evinced by measuring LC3B. Compared to the MSCs in the untreated control group which showed reduced autophagy, the cells treated with AICAR, NAM, and combined AICAR+NAM displayed upregulated autophagy, with the latest having the highest rate of autophagy (Fig. 5a, b). Altogether, while aging is associated with decreased autophagy, inhibiting mTORC1 by upregulation of active AMPK through the use of AICAR and NAM can revert the effect of aging and lead to an increased rate of autophagy.

## Discussion

To determine the impact of administration of AICAR and NAM on in vitro aging of MSCs, we designed and performed a series of experiments. Doubling time and MTT assay were performed to indicate the proliferative capacity of each group at different time points of the culture. We observed that while the doubling time of the control MSCs gradually increased, all the three treatment groups had lower doubling times at all time points, especially at the P10. Additionally, after 7 days of culturing the P10 cells, all the three treatment groups had a higher cell density compared with the control MSCs, indicative of faster proliferation and longer preservation of proliferative capacity. It is important to note that AICAR only- and NAM only-treated cells were not significantly different in their MTT assay; however, MSCs concomitantly treated with both had a significantly higher proliferative capacity. This may indicate a synergism or additive effect between AICAR and NAM. Moreover, we observed a decline in the expression of cyclin-dependent kinase inhibitor genes—*P16* and *P21*—that regulate the G1 to S phase transition. MSCs treated with AICAR+NAM showed a reduction in the expression of *P16* and *P21*. In support of our data, Gharibi et al. [11] observed that MSCs treated with rapamycin showed a significant decline in the expression of *P16* and *P21* mRNAs.

According to the literature, AICAR affects the cell growth and proliferation capacity in a dose- and cell type-dependent manner [26, 34, 35]. Wu et al. demonstrated that 1 mM AICAR inhibited the growth of human amniotic MSCs and rabbit bone marrow-derived MSCs. They further showed that concentrations as low as 0.1 mM increased the proliferation of Amniotic MSCs even further, while still slightly inhibiting the growth of the rabbit MSCs [26]. Additionally, whereas 0.5 mM AICAR tripled the Caspase-3-positive cells in mouse embryonic stem cell culture, it increased the cell cycle progression to the extent that the net proliferation was higher than the controls [34]. It is also not surprising that we observed a greater proliferation capacity in our AICAR-treated group, as Shi et al. [35] demonstrated that 1 mM concentration of AICAR could sustain mouse embryonic stem cell self-renewal although most studies showed that 1 mM concentration of AICAR inhibited the growth of the cultured cells [26, 34].

Morphologically speaking, our data showed that AICAR could prevent the morphological features of senescence, whereas NAM lacked this ability. Additionally, all three treatment groups had a lower frequency of SA-β-gal-positive cells per equal area of the culture dish. Again, AICAR- and AICAR+NAM-treated cells had an even lower frequency of SA-β-gal-positive cells compared with the NAM-only treated group.

When stained with Acridine Orange, all of the treatment groups had a statistically equal number of senescent cells (those that emit green fluorescence) per equal number of cells counted in each culture flask, which was significantly less than that of the control group. When treated with Acridine Orange, double-stranded DNA emits green fluorescence, while acidic vesicular organelles, like lysosomes in their intact and functional form, emit a distinctive reddish-orange fluorescence. Interestingly, when the lysosomes lose their acidity, they also change their color to green. It has been proven that the activity of the lysosomes and acidity of these vesicular organelles are correlated with the level of autophagy; the greater the number of red dots in a cell is, the higher the autophagy level would be [1, 30, 36]. Senescent cells often exhibit a characteristic accumulation of dysfunctional autophagolysosomes, portrayed as a yellowish-green granulated cytoplasm when treated with Acridine Orange [1, 30, 36], as seen in the control group. Altogether, our data is consistent with the literature [11, 24], as treatment with senolytics AICAR and NAM, especially AICAR, prevented most of the morphological features of senescence in our MSCs, as shown by smaller cell surface area, lower frequency of SA-β-gal-positive cells, and accumulation of autophagolysosomes in the cytoplasm.

Comparing the AICAR group with the controls, we observed that they were not statistically different in terms of the ROS level. Based on the free radical theory of aging [19, 37, 38], these two cells must show similar senescence-associated changes like the morphology and the molecular markers. On the contrary, the AICAR group had a significantly lower surface area, almost a third of that of the control group. Additionally, it has a much fewer number of cells that are positive for the SA-β-gal and dysfunctional lysosome accumulation in the cytosol. The AICAR group also had a higher level of active AMPK and LC3B level and a lower level of mTORC1 activity.

Comparing the NAM group with the controls, we found that the NAM group had a significantly lower level of cytosolic ROS. However, unlike the other groups, NAM was not significantly different from the controls in terms of the surface area. While many are on the belief that free radicals are the cause of aging, or at least they are the source of aging-associated damages [19, 37], our data indicated that the ROS level was not associated with the aging of MSCs. It is shown that both AMPK signaling and sirtuins could decrease the level of ROS [39], but it is not clear why AICAR could not significantly decrease free radicals in our study.

Anyhow, it is not the first time that the role of ROS in aging, cancer, and cell biology is undermined [38]. It has been shown that the reduction of free radicals by *N*-acetyl cysteine not only did not improve the overall survival but also further intensified the metastasis of the tumor cells in a murine model of melanoma [40] and lung cancer [41]. Additionally, reevaluation of clinical efficacy of antioxidants in a meta-analysis revealed that micronutrients, vitamins, and antioxidants were only effective in individuals with levels below the minimum required quantity but not in those with levels above that [42]. Moreover, Zhu et al. demonstrated that cellular ROS level is not even correlated with its cytotoxicity [43]. Finally, it is shown that while vitamin C and *N*-acetyl cysteine reduce the ROS level, both in vitro and in vivo, they have limited efficacy in terms of geroprotection, especially in vivo [17, 44].

Consistent with the literature [11], we observed that the inhibition of the mTORC1 pathway reversed the adverse effects of prolonged culture on the osteogenic differentiation capacity of MSCs. Interestingly, our data demonstrated that concomitant use of AICAR+NAM resulted in an even more induction of osteogenesis, indicating an additive or synergistic effect of these two compounds on osteogenic properties of MSCs.

While we detected a greater level of lipid accumulation in the treatment groups, the expression of LPL and PPAR-γ mRNAs were decreased after treatment with AICAR and NAM. We think this is because treatment with AICAR and NAM can increase the proliferation of the cells, which might show a greater level of lipid accumulation in the treatment groups after Oil Red O staining. In line with our findings, Gharibi et al. previously showed that while treating MSCs with rapamycin resulted in an increased level of lipid accumulation in the cells, it had an insignificant effect on the markers of adipogenesis [11]. Additionally, as demonstrated by Jaiswal et al., differentiation of MSCs to the osteogenic and adipogenic lineages is mechanistically related [45]. Together, these findings propose an inverse relation between osteogenic and adipogenic differentiation of MSCs in aging [11].

When treated with either AICAR, NAM, or their combination, the frequency of Bcl-2-positive cells increased and that of Bax- and Caspase-3-positive MSCs dropped significantly. Flow cytometry analysis showed that AICAR-treated cells had the highest while NAM-treated cells had the lowest level of ANNEXIN-positive cells, even less than a third of the AICAR-treated MSCs. This probably happens because there is a one-way molecular switch between autophagy and apoptosis. Tavassoly et al. developed a computational model and described autophagy as a rheostat that tolerates the stressors; if autophagy is unable to counteract the damages, then apoptosis terminates that irreparable cell [46, 47]. Since AICAR increases autophagy, it also enhances the apoptotic fraction of cells, as Grigorash et al. seen in AICAR-treated embryonic stem cells [34].

**Fig. 6:**
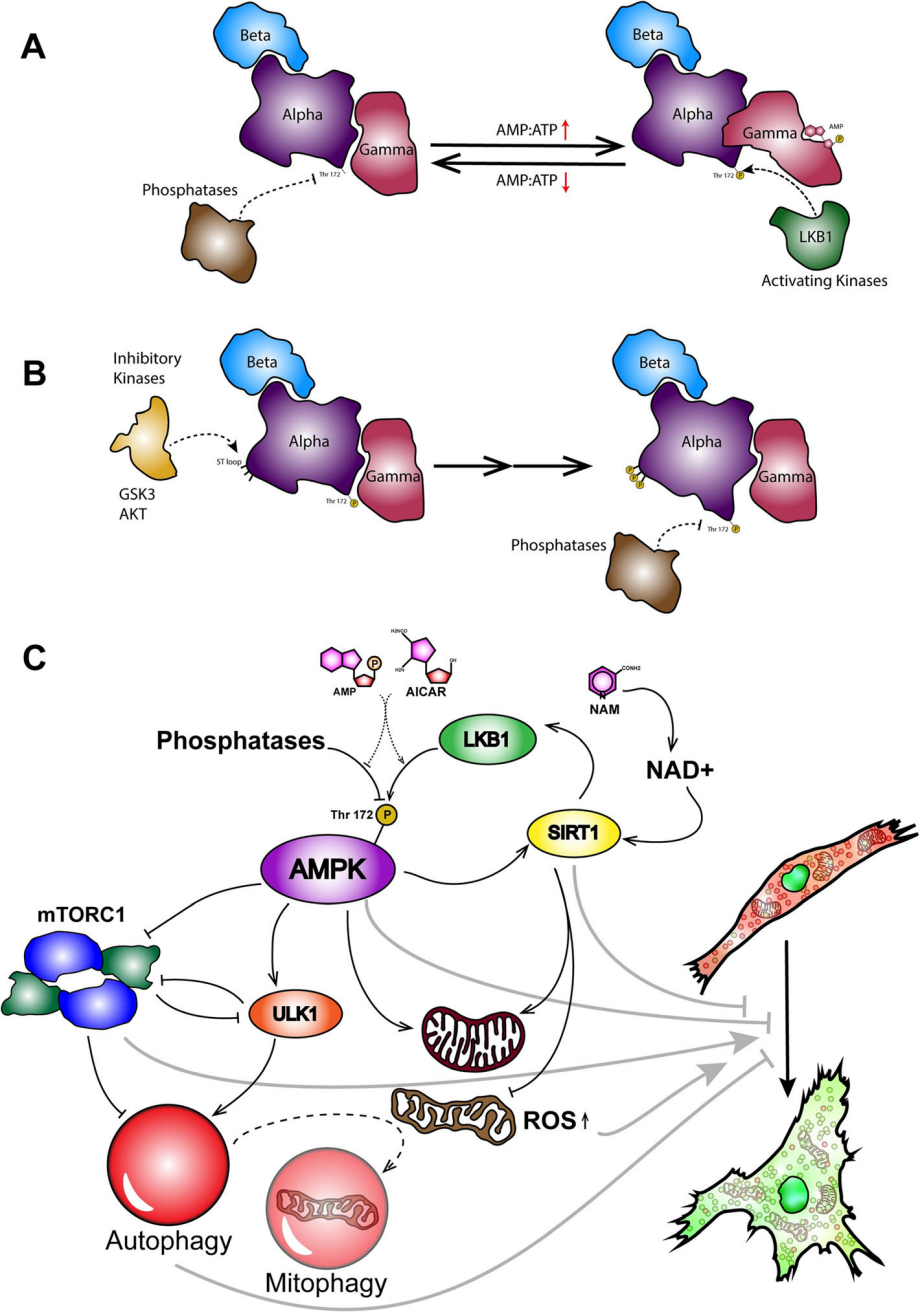
The schematic mechanism of action of AICAR and NAM. **a** AMP binds to the gamma subunit of AMPK, allosterically increases the exposure of T172 to the activating upstream kinases like LKB1 and CaMKK-beta, and hinders the access of upstream phosphatases like PP2A to this residue. **b** AMPK may undergo a series of phosphorylations by inhibitory upstream kinases like GSK3, AKT, and ERK; GSK3 phosphorylates the ST loop of the alpha subunit and primes the subsequent phosphorylations for others. Later on, the alpha subunit changes its conformation, and the T172 gets exposed to the inhibitory phosphatases. **c** Acting like AMP, AICAR increases T172 phosphorylation of the alpha subunit by facilitating the phosphorylation and hindering the dephosphorylation. Following that, AMPK activates autophagy in general. Additionally, AMPK increases the biogenesis of new mitochondria and the degradation of dysfunctional and damaged ones. Moreover, NAM increases SIRT1 activity. In turn, SIRT1 increases the LKB1 activity and indirectly increases the AMPK activity. Furthermore, it increases the functionality of mitochondria and decreases ROS production by damaged mitochondria. These sequence of events prevents the MSCs to age and to show senescence-associated changes like dysfunctional autophagosome accumulation, and enlarged and flattened morphology

Our results showed that the senescent MSCs had a much lower level of autophagy determined by Acridine Orange and immunofluorescence staining of LC3B. AICAR efficiently increased the AMPK Thr172 phosphorylation and decreased the mTORC1 activity; this led to an increase in autophagy. This preserved the proliferation capacity and reduced the cell size in the tested MSCs. Moreover, solely the NAM treatment significantly increased autophagy and AMPK activity and decreased the mTORC1 activity, but not as well as AICAR-alone treatment. Furthermore, NAM could not preserve the proliferative capacity and size attributes of MSCs, as efficient as AICAR does. However, as NAM-mediated activation of SIRT1 increases the mitochondrial biogenesis and homeostasis, as well as LKB1 activity, it is predictable to see a reduction in cellular ROS and increased level of anti-apoptotic Bcl-2, probably by increased mitochondrial stability, and also a slight elevation of autophagy. Last but not the least, AICAR+NAM treatment showed a synergism, or at least an additive effect, toward the prevention of cellular senescence. In fact, AICAR+NAM-treated cells showed the beneficial effects of both AICAR and NAM (Fig. 6).

AMPK is the evolutionarily conserved master energy sensor/regulator of the eukaryotic cells [23, 48]. It consists of three subunits, namely alpha, beta, and gamma, among which alpha is the kinase subunit, and the other two are the regulatory ones [48, 49]. AMPK senses the AMP to ATP and ADP to ATP ratios to maintain the available energy for cellular functions above a tightly controlled threshold. AMP and ADP allosterically bind to the gamma subunit to simultaneously increase the access of activating kinases and restricting the access of phosphatases to the Thr172 of the alpha subunit [48, 49]. LKB1 is the main upstream kinase that phosphorylates the activatory Thr172 site [48, 49]. Other phosphorylation sites are also the allosteric regulators of the Thr172; to be specific, glycogen synthase kinase 3 (GSK3) phosphorylates the Thr479 of the alpha subunit to prime its phosphorylation by other inhibitory kinases, namely AKT (protein kinase B), PKA (protein kinase A), and ERK (extracellular signal-regulated kinase) in the serine-threonine-rich (ST) loop of the alpha subunit. These consecutive phosphorylations increase the sensitivity of the Thr172 to its phosphatases [48–50]. AICAR, which is the analog of AMP, binds to the same site on AMPK and activates it by mimicking the energy deprivation that is normally determined by AMP to ATP ratio (Fig. 6a, b) [26, 49, 51].

Another important species that interacts with AMPK is SIRT1 [48, 49, 52]. AMPK and SIRT1 form a very interesting positive feedback loop; AMPK activates SIRT1 via several mediators to control the mitochondrial biogenesis and expression of some anti-stress genes. Additionally, NAM increases the level of NAD^+^ and activates many NAD^+^-dependent processes, including SIRT1. Activation of SIRT1 upon NAM treatment increases the expression level of many stress-attenuating species and augments the LKB1/AMPK axis activity [49, 52–56]. In short, we hypothesized that the AICAR+NAM synergism was mainly due to the simultaneous allosteric activation of AMPK by AICAR and augmented LKB1 activity by NAM (Fig. 6a–c).

Many of the processes that are under control of AMPK are involved in the pathogenesis of aging, among which, perhaps, autophagy is the most important one [48, 49]. There is a body of evidence showing that aged cells have reduced autophagy and AMPK activity. It has also been demonstrated that suppressed autophagy is the main factor in the pathogenesis of aging in several tissues and cell types, especially in stem cells [18, 48, 49, 57, 58]. For instance, García-Prat et al. showed that treating old mice with a rapamycin regimen increased autophagy and re-established cell proliferation [18]. Additionally, Ok et al. demonstrated that human bone marrow MSCs treated with NAM had increased autophagy [24]. According to Morselli et al., increasing autophagy is an integral part of the geroprotection, and without it, senolytics would not show any apparent protection against aging [59]. Autophagy is mostly under the control of the kinase triad of mTORC1/ULK1/AMPK [60, 61]. ULK1 is the autophagy-activating kinase that derives autophagosome formation and activation by mediating LC3B conversion. mTORC1 and ULK1 mutually inhibit each other to temporarily separate the two irreconcilable processes of protein synthesis and autophagy, respectively. AMPK activates ULK1 to derive the cell toward autophagy. It also inhibits mTORC1 through both phosphorylations of its upstream, TSC1/2 complex, and by phosphorylating Raptor, a regulatory component of mTORC1. As mTORC1 is an autophagy suppressor, its inhibition by AMPK is yet another mechanism to activate autophagy [18, 48, 60, 61]. Two processes are involved in the reduction of AMPK activity during aging. Hua et al. and Zhang et al. showed that in some tissues, like myocardium, the basal phosphorylation of Thr172 on the alpha subunit significantly drops in aged individuals [57, 62]. Additionally, it has been demonstrated that while there is no difference between the aged and young levels of Thr172 in other cells, the responsiveness and sensitivity of this phosphorylation site drop drastically in aged animals, regardless of the tissue or cell type. This decreased sensitivity and phosphorylation level is attributed either to the increased action of the corresponding phosphatases or to the decreased activity or access of upstream kinases on this residue [49, 51, 58, 63]. One explanation would be the age-related mitochondrial defects, leading to a gradual disturbance of energy balance and AMP level [48, 49]. In turn, AMPK cannot be efficiently activated anymore, causing a gradual decline in autophagy. An additional reduction of autophagy leads to the accumulation of dysfunctional mitochondria, which also increases the cellular ROS level. These are only a small part of the vicious cycle that eventually leads to cellular senescence [48, 49, 53–55, 64, 65].

Our study faces several limitations. Here, we did not analyze the effects of AICAR and NAM treatment on cell cycle and whether or not each compound, or their combination, can cause dysregulation in the cell cycle. This weakens our results regarding the effects of AICAR and NAM on the proliferation of the MSCs. In addition, we did not evaluate the effect of AICAR and NAM on the secretome of the MSCs after prolonged in vitro culture. Furthermore, while we studied the effects of AICAR and NAM on the mTORC1 signaling pathway, we did not evaluate how each compound and their combination might affect the mTORC2 signaling pathway.

## Conclusion

In summary, to delay the senescence, increasing autophagy by selective inhibition of mTORC1 seems to be an efficient approach to employ. Increased autophagy cleans up the cell from damaged organelles, like mitochondria. Additionally, mTORC1 inhibition keeps the cells sensitive to growth factors and preserves their proliferative capacity while in culture; this will help us have a higher number of young cells after in vitro expansion. Not to forget that future studies are needed to investigate the effects of mTORC1 inhibition on in vivo geroprotection/rejuvenation. Furthermore, our results raise a question on the role of ROS as a key player in the pathogenesis of in vitro aging of MSCs.

## Supplementary information

**Additional file 1.** Effect of AICAR+NAM treatment on mRNA expression of *P16* and *P21.* Analysis of mRNA expression of *P16* and *P21* determined by qRT-PCR. (*n=*3 independent experiments). Each bar indicates mean ±SD.

## Data Availability

The data that support the findings of this study are available from the corresponding author upon reasonable request.

## Abbreviations

MSC: Mesenchymal stromal cell
AICAR: 5-Aminoimidazole-4-carboxamide ribonucleotide
NAM: Nicotinamide
SIRT1: Sirtuin1
P: Passage
ROS: Reactive oxygen species
mTORC1: Mechanistic target of rapamycin complex 1
AMPK: 5′-Adenosine monophosphate-activated protein kinase
Raptor: Regulatory-associated protein of TOR
PBS: Phosphate-buffered saline
DMEM: Dulbecco’s modified Eagle’s medium
FBS: Fetal bovine serum
MTT: 3-(4,5 Dimethyl-2-thiazolyl)-2,5-diphenyl tetrazolium bromide
OD: Optical density
SA-β-gal: β-Galactosidase
DCFDA: 2′,7′-Dichlorofluorescin diacetate
Runx-2: Runt-related transcription factor 2
ALP: Alkaline phosphatase
qRT-PCR: Quantitative reverse transcription polymerase chain reaction
LPL: Lipoprotein lipase
PPAR-γ: Peroxisome proliferator-activated receptor-γ
DAPI: 4,6-Diamino-2-phenylindole dihydrochloride
S6K1: S6 kinase 1
GSK3: Glycogen synthase kinase 3
AKT: Protein kinase B
PKA: Protein kinase A
ERK: Extracellular signal-regulated kinase
