# Supplementary material for: AICAR and nicotinamide treatment synergistically augment the proliferation and attenuate senescence-associated changes in mesenchymal stromal cells
Source: Stem Cell Res Ther. 2020 Feb 3;11:45. doi: 10.1186/s13287-020-1565-6 (PMC6998366; doi:10.1186/s13287-020-1565-6)

Additional file 1. Effect of AICAR+NAM treatment on mRNA expression of *P16* and *P21.* Analysis of mRNA expression of *P16* and *P21* determined by qRT-PCR. (*n=*3 independent experiments). Each bar indicates mean ± SD.


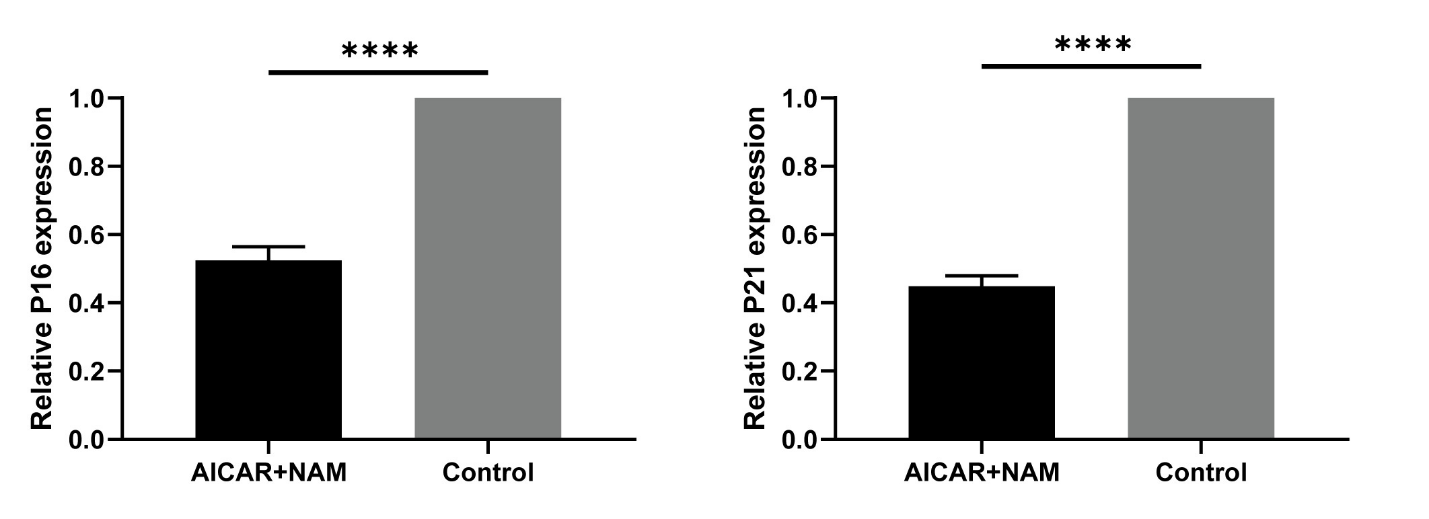

Supplement: Supplementary file 1 — Additional file 1. Effect of AICAR+NAM treatment on mRNA expression of P16 and P21. Analysis of mRNA expression of P16 and P21 determined by qRT-PCR. (n=3 independent experiments). Each bar indicates mean ±SD. [file 13287_2020_1565_MOESM1_ESM.docx]
